# Interventional Pain Management: Need of the Hour for Cancer Pain Patients

**DOI:** 10.4103/0973-1075.58451

**Published:** 2009

**Authors:** Sushma Bhatnagar

**Affiliations:** Editor, IJPC, Associate Prof. and Head Anesthesiology, Department of Anesthesiology, Pain and Palliative Care, Dr. B.R. Ambedkar, IRCH, AIIMS, New Delhi, India E-mail: sushmabhatnagar1@gmail.com

Approximately 28% of patients with cancer pain die with severe untreated pain despite effective multidisciplinary techniques that should treat these patients effectively.[1] Despite the public fear that cancer is always linked with severe pain, majority of the cancer patients can have pain adequately controlled with standard analgesics administered on a regular basis as described by the World Health Organization (WHO) treatment guidelines for cancer pain.[2] Clinical trials throughout the world demonstrate that most patients (85%-90%) with cancer pain are well managed using traditional opioid and non-opioid analgesics via the oral, transdermal, or parenteral route.[3] One-third of cancer patients experience pain at the time of diagnosis and 70% experience painful symptoms during the advanced stages of their disease.[4] Clinical analgesic studies of patients with cancer also demonstrate that approximately 10-15% of patients experience severe pain that is resistant to traditional analgesic therapies.[5] For this significant minority of patients, resistance to traditional analgesics or pain relief is achieved only at the expense of severe side effects; selective interventional analgesic treatment options must be considered. Interventional approaches do work and many authors propose the addition of interventional strategies to the WHO guideline as a fourth step of this ladder.[6]

Interventional pain management procedures are most often used in concert with standard analgesic regimens to reduce opioid side effects or gain better analgesic efficacy. The interventional analgesic technique may be used at any time during the course of cancer treatment but is often employed during the more aggressive phases of disease.[7] Interventional techniques for cancer pain management are regarded as part of a multimodal approach to pain relief and not as a stand-alone therapy.[8] Interventional pain management for patients with cancer generally falls into one of two management categories: Surgical or anesthetic. The use, indications, efficacy, and timing of appropriate surgical management of cancer pain have been well reported by Hassenbusch.[9] The use of destructive techniques, through surgery or anesthetic block, has decreased in recent years with the advent of nonablative measures such as local anesthetic and opioid infusions. The use of local anesthetic infusions for the management of cancer pain has been successful with peripheral somatic nerve plexus blockade (e.g., brachial plexus catheter for arm pain) and epidural and subarachnoid infusions for the control of pain of the chest wall, abdomen, back; or lowers extremities, to name a few examples.[7] The recent use of ultrasound imaging for nerve localization is an innovative application of an old technology, which addresses many of the shortcomings of current techniques.[10] The aggressive treatment of cancer pain may even be considered good oncologic practice, since there is some evidence that uncontrolled pain contributes to a shortened life expectancy.[11] Hence, every effort should be made to treat all cancer pain patients with all the available interventional techniques if the conventional techniques failed. It is the hour of need for evolution of palliative medicine into “interventional palliation” with goal to strengthen the physical arm of the palliation and balance the “psycho-social-spiritual” arm of holistic approach.[12] A wide array of procedures exists (e.g., local anesthetic/steroid deposition, neurolysis by chemical or thermic means, or the implantation of spinal pumps to deliver medications not effective by the oral/transcutaneous route) that have their own indications and side-effects profile. The pain practitioner, interventionalist or not, needs to be aware of the various options in order that an appropriate choice for comfort may be made.[13]

Before jumping into interventional technique, patients should receive appropriate trials of opioid and non-opioid analgesics. A complete pain evaluation with pain history, appropriate physical examination (including a neurologic examination), and a tentative etiology for the pain is necessary prior to any invasive procedures. A neurologic examination identifies any preexisting neurologic deficits as well as areas of minor motor or sensory reduction, Informed consent is a prerequisite, especially with the neurolytic blocks that may result in permanent motor/sensory deficits. A local anesthetic block is usually performed prior to any “permanent” neurolytic procedure to evaluate the likelihood of success and identify neurologic deficits that may be intolerable.[7]

Virtually all studies, to date, recommend the proper selection of patients so that less invasive analgesic techniques may be utilized as first-line therapy.

The wide variety of interventional pain management techniques available in the present situation should encourage physicians to consider “something further” in almost all cases of cancer pain. The appropriate use of anesthesiologic interventions to help manage cancer pain will improve the quality of life for all patients.

There is a need for research to determine clear indication and benefit from interventional pain management techniques in cancer pain patients.
